# Niche partitioning and host specialisation in fish‐parasitising isopods: Trait‐dependent patterns from three ecosystems on the east coast of India

**DOI:** 10.1002/ece3.70298

**Published:** 2024-09-11

**Authors:** Sandeep Kumar Mohapatra, Anshuman Swain, Dipanjan Ray, Rajesh Kumar Behera, Basudev Tripathy, Jaya Kishor Seth, Anil Mohapatra

**Affiliations:** ^1^ Estuarine Biology Regional Center, Zoological Survey of India Ganjam India; ^2^ Post Graduate Department of Zoology Berhampur University Berhampur India; ^3^ Department of Biology University of Maryland College Park Maryland USA; ^4^ Department of Organismic and Evolutionary Biology Harvard University Cambridge Massachusetts USA; ^5^ Museum of Comparative Zoology Harvard University Cambridge Massachusetts USA; ^6^ Department of Zoology Bajkul Milani Mahavidyalaya Purba Medinipur India; ^7^ Western Regional Centre, Zoological Survey of India Pune India

**Keywords:** bipartite network, cymothoid isopods, fish parasites, host–parasite interactions, trait‐based analysis

## Abstract

Due to their large size and obligate nature, Cymothoid isopods inflict a high degree of tissue damage to fish. Still, they are understudied at an ecosystem level despite their global presence and ecological role. In this work, we collected fish host‐isopod parasite data, along with their life history and ecological traits, from the northern part of the east coast of India and investigated patterns in host specialisation and preference of isopod parasites using a trait‐based network perspective. We observed that the region of attachment of the parasite (buccal cavity, branchial cavity, and skin) and host fish ecology (schooling behaviour and habitat characteristics) influenced host specialisation and preference. We found that branchial cavity‐attaching parasites preferred schooling, pelagic fishes, whereas buccal cavity‐attaching parasites preferred mostly non‐schooling, demersal fishes. Skin‐attaching parasites were found to be generalists and had no preference based on our examined host traits.

## Graphical abstract

The fish‐parasitic isopods of family cymothoidae have different preferences for hosts according to their traits viz., demersal, pelagic, or according to their habitat in the water column.

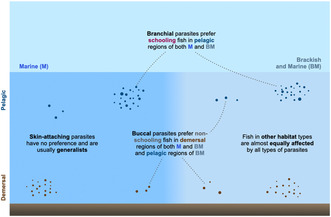

## 1 INTRODUCTION

Parasites account for over 40% of the faunal diversity in ecosystems (Hatcher and Dunn, 2011), and in many cases, make up the majority of animals in aquatic systems (Sikkel & Welicky, 2019). By definition, they adversely impact the survival and reproduction of host organisms, but the extent to which they have community and population‐level impacts (such as changes in organismal fitness, individual and group behaviour, and the life‐cycle structure of their hosts) is poorly understood and depends on the interplay of a variety of environmental factors and the degree and pervasiveness of the host–parasite association (Sikkel & Welicky, 2019). This lack of knowledge becomes crucial when dealing with host organisms of combined ecological, social, and commercial importance, such as fish.

Fish play a major role in aquatic ecosystems, by controlling the populations of other organisms through predation, mediating nutrient fluxes, and acting as ecosystem engineers (Villéger et al., 2017). They also serve as a major source of sustenance for a large proportion of humans globally (Chan et al., 2019; Hasselberg et al, 2020). Therefore, understanding the impact of various parasites on fish populations is important for both ecosystem functioning and ecosystem services rendered to humans (fisheries). Isopods from the family Cymothoidae are often larger than other fish parasites (Smit et al., 2014). Their larger size and obligate nature lead to a greater degree of tissue damage, inflammation, and constant irritation in their fish hosts (Purivirojkul & Songsuk, 2020). These isopods attach to different parts of the host's body: the buccal cavity, the branchial cavity, and the skin surface (Jones et al., 2008). Some species even bore into the host skin and live inside the skin pouch (Smit et al., 2014). Unlike their sister isopod families, such as Aegidae or Gnathiidae, which are temporary parasites and detach from their hosts after a blood meal (Bruce, 2009; Tanaka, 2007), the Cymothoids spend their complete adult life attached to the host and feeding on blood, mucus, and flesh (Mahmoud et al, 2016; Rameshkumar & Ravichandran, 2014).

Past studies have suggested that host specialisation (i.e., number of impacted host species) in these isopods depends upon the mode of attachment to the host; however, these studies included only a few host/parasite species and have not been performed at an ecosystem scale (Morton, 1974). Even within comparable habitats of hosts, isopods with the same attachment type can vary in their host specificity, e.g., many buccal‐attaching isopods are known to infect a single host; for example, *Lobothorax typus has been* reported from *Trichiurus lepturus* Linnaeus, 1758; *Cymothoa bychowskyi* Avdeev 1979 is known to infect *Fistularia petimba* Lacepède, 1803; *Ceratothoa angulata* (Richardson, 1910) has been reported from *Hyporhamphus dussumieri* (Valenciennes, 1847) (Rameshkumar et al., 2013, 2014; Ravichandran et al., 2019) whereas another buccal parasite, *Cymothoa indica* Schioedte and Meinert, 1884 is reported from fishes belonging to the seven distinct families (Chilton, 1924; Mohapatra et al., 2022; Panikkar & Aiyar, 1937; Rajkumar et al., 2005; Ravi & Rajkumar, 2007; Ravichandran et al., 2010; Veerapan & Ravichandran, 2000). Thus, in addition to the parasite mode of attachment to the host, these patterns will also be mediated by the habitat (e.g., marine or brackish waters; demersal or pelagic habitats) and host life history (Smit et al., 2014). Therefore, exploring the patterns of specialisation and preference among these parasites and the factors, both ecological and organismal, that affect the presence of a host–parasite association is important to understand the impact of these parasites on fish populations and how these interactions impact the ecosystems at large.

**FIGURE 1:**
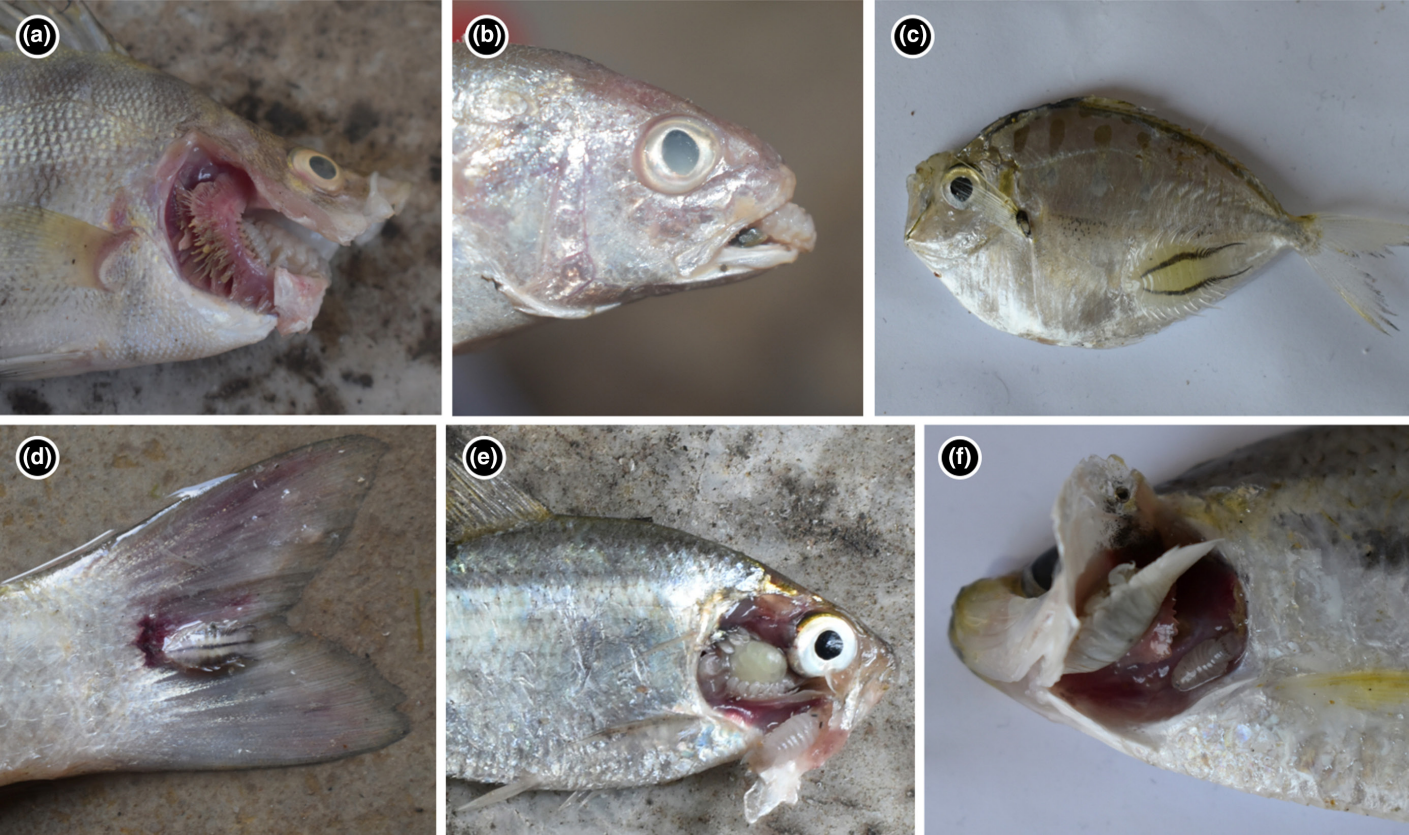
Fish–parasite interactions from the east coast of India: (a) *Cymothoa indica* male (smaller one in the sub‐panel) and female attaching to the buccal cavity of *Datnoides polota*; (b) *Cymothoa indica* attaching to the buccal cavity of *Jonhius* sp.; (c) *Nerocila loveni* attaching to the skin in the ventrolateral region of *Deveximentum Interruptum*; (d) *Nerocila orbignyi* attaching to the tail skin of *Mugil cephalus*; (e) *Agarna malayi* attaching to the branchial cavity of *Nematolosus nasus*; (f) *Joryma sawayah* male (smaller one in the sub‐panel) and female attaching to the branchial cavity of *Nematolosus nasus*.

In this work, we collected and compiled detailed data on Cymothoid isopods parasitic on fish from multiple sites along the east coast of India (see Figure 1 for details), along with functional trait data about both host fish species and parasites. We applied techniques from network analysis to disentangle aspects of host specialisation and niche partitioning among the parasites based on their trait data. Past work using network analysis in the context of fish parasitism (Runghen et al., 2021) has assessed the eco‐evolutionary impacts of parasites on different levels of biological organisation such as evolutionary immune response to parasitic infections (Pilosof et al., 2014), enemy release in invasive hosts (Llopis‐Belenguer et al., 2020), modularity of host–parasite associations (Hiller et al., 2021), changes trophic structure and efficiency in food webs (Arias‐González & Morand, 2006), etc. The detailed network datasets used in past studies, despite covering many phyla of parasites, do not include many isopods and even if they do, most of them are not identified at a species level and are primarily skin‐attaching parasites (see Poulin and McDougall, 2022). Almost all of these datasets are from the Americas, barring a few from Eastern Europe and Vietnam, and one from Bangladesh (which has just one isopod species) (Poulin and McDougall, 2022). Therefore, the current study also fills geographical, taxonomic, and ecological gaps in the available data on fish–parasite interactions. In addition, we go beyond previously used network analyses, and perform novel node‐level and link‐level network analyses, based on the traits of both parasites and host species, and investigate patterns of specialisation and niche partitioning among these isopods.

## 2 METHODS

### 2.1 Data collection

Fish and their associated parasitic isopods were collected from different fish landing centres and fishing harbours along the eastern coast of India (specifically, the states of Odisha and West Bengal) between 2015 and 2022. The branchial cavity, buccal cavity, and body surface of the fish were checked for the presence of parasites. The attachment sites of parasitic isopods on the host fish were recorded, and the collected parasites were photographed and preserved in 90% ethanol for further examination.

**FIGURE 2:**
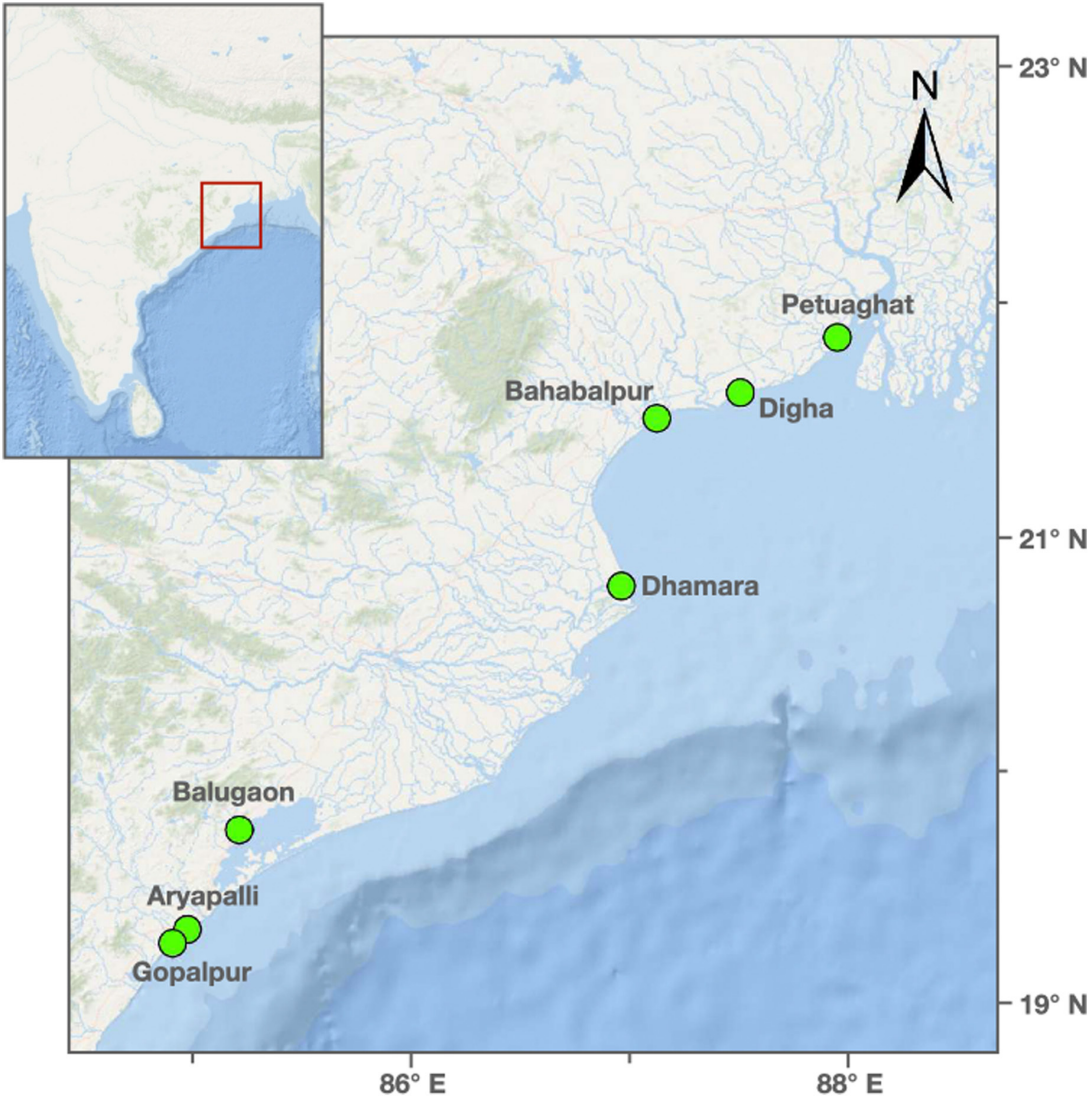
Sampling sites: 7 sites (denoted by green dots on the larger map) were sampled along the east coast of India (in the states of Odisha and West Bengal). The inset shows a larger geographical context of the region of interest.

In specific, the landing centres of Petuaghat and Digha in West Bengal, and Bahabalpur (and nearby Balaramgadi), Dhamara, Balugaon, Gopalpur, and Aryapalli in Odisha were targeted (see Figure 2). The sites had a depth of less than 3 km at the operational fishing catchment areas along the coast where data were collected for this work. The fish were landed using hook nets (longline nets) and bottom trawling far from shore for catching fish in the deeper sea (within 50 km of the coast in all cases). Dragnets and gill nets were used for catching fish near the shore. Cast nets and scoop nets were used in the estuary region. The fish were mostly checked in the trawler itself, and to a lesser extent at the fish landing centres, and in both cases, there were commercial and non‐commercial fish present (i.e., random sampling, none discarded). As the fish were captured with a variety of methods and sampled randomly, and the methods were consistent across all the sites, we expect low sampling bias within and among sites.

In total, 5798 fishes were examined from 120 species, out of which 923 (from 59 species) were found parasitised by 1038 Cymothoid isopods (from 21 species) (see Table S5 for details). The parasitic isopods were identified by using standard keys and descriptions provided by Ravichandran et al. (2019); Roy et al. (2024) and the host fishes were identified using Talwar and Jhingran (1991) and Froese and Pauly (2024). The specimens were deposited in the National Zoological Collections at the Estuarine Biology Regional Centre (EBRC) of the Zoological Survey of India (Gopalpur‐on‐sea, Ganjam, India).

We also assembled secondary metadata describing the functional traits of the host fishes and the attachment site information for the isopods. The fish functional traits included (1) habitat salinity (either marine, brackish‐marine, or brackish; the ones categorised as brackish‐marine are found in both environments); (2) schooling behaviour (either schooling or non‐schooling); and (3) habitat zone (either pelagic, demersal or benthic) (Mbaru et al., 2020).

### 2.2 Sampling completeness and traditional knowledge

Even though we had collected past literature to inform our sampling procedure, we independently leveraged the knowledge of local fisherfolk communities to enumerate all possible fish hosts containing parasitic isopods (please note that this did not impact our sampling regime at all). Validating their knowledge using modern techniques was a unique aspect of our project and also allowed us to check for the lack of any obvious host fish/parasites known to traditional fishing communities. We found that their information was very accurate when compared with our sampling efforts.

To statistically test for sampling completeness of the parasite community and the host–parasite interactions, we calculated the sampling coverage for the two sampled regions using the iNEXT package in Rv4.2.2 (Hsieh et al., 2016; R Core team, 2023; see Nikkeshi et al., 2021 for similar usage in pollen networks; Swain et al., 2024 for biogeographic networks).

### 2.3 Statistical analyses

**FIGURE 3:**
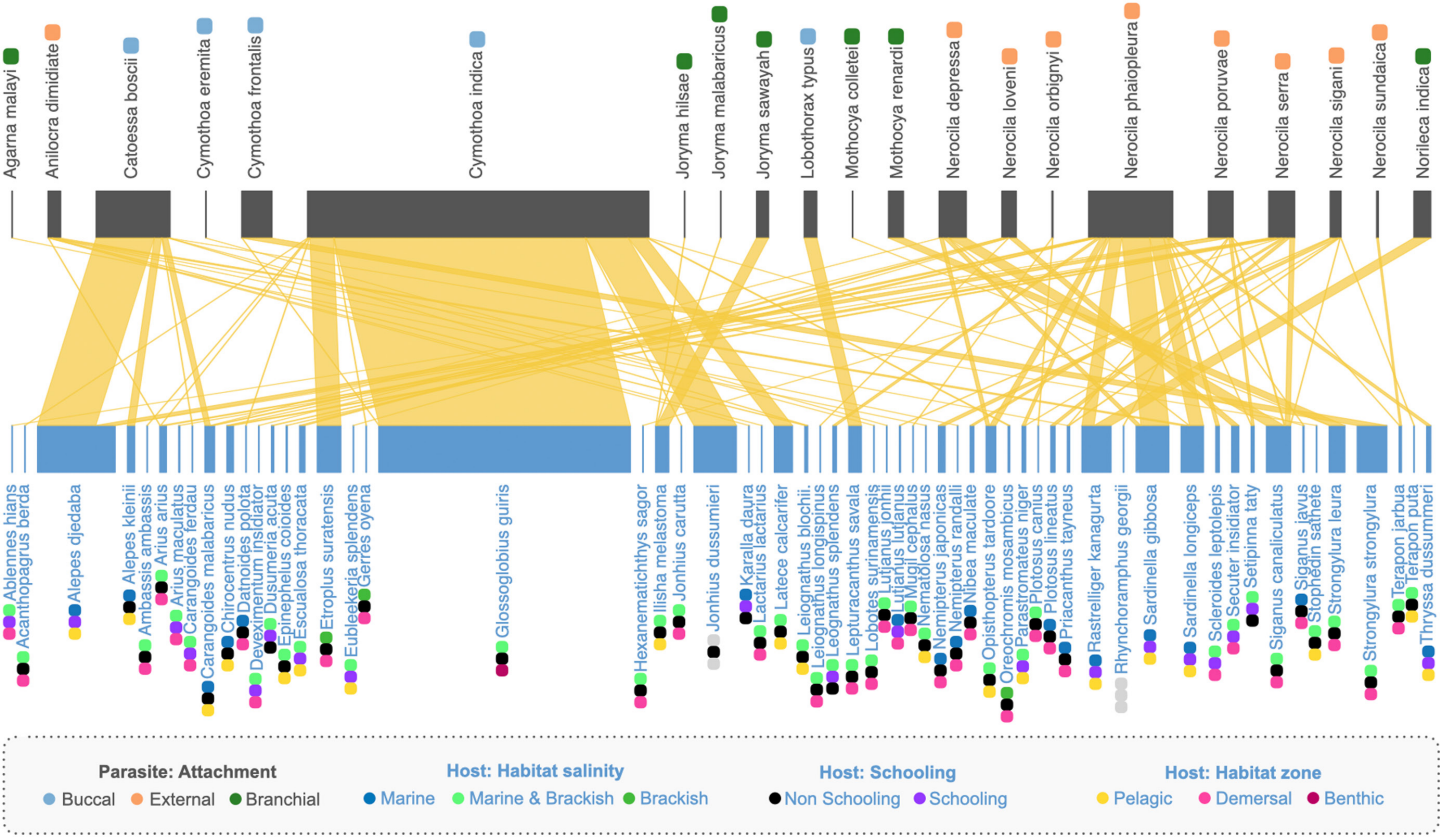
Fish–parasite networks: Representations of weighted fish host‐isopod parasite interactions from the Eastern Coast of India, along with functional traits of both the fish host (habitat salinity, schooling behaviour, and habitat zone) and the isopod parasite (attachment site). The thickness of the lines depicts the relative number of parasites found in different host fish species.

Once sampling coverage over 95% was established for the data, the data were analysed using the R language v4.2.2 (R Core team, 2023). We first constructed a bipartite network (defined as networks with two classes of nodes and where links can exist only between nodes of different classes) with fish hosts and isopod parasites as the two node classes (Figure 3) (Runghen et al., 2021). Each species denoted a node and a link/edge was drawn between a host (node) and a parasite (node) to show the presence of an association. We computed the following network metrics at the (parasite) node level using the R package bipartite (Dormann et al., 2009): (1) degree (number of host species), (2) effective number of hosts (ENH; number of ‘effective’ host species weighted and corrected for variation in the number of hosts infected per species of the host (see Bersier et al., 2002; Dormann et al., 2009)), (3) and (4) host specificity index (HSI) and host paired different index (HPDI) (both signify the variation in the number of hosts infected across different host species with 0 denoting very generalised interactions and 1 denoting extreme specialisation (Poisot et al., 20,211, 2012); HSI is based on the species specificity index (Poisot et al., 2012, Woodhouse et al., 2023)). We tested the hypothesis posed in earlier literature (Morton, 1974) about skin‐attaching parasites being generalists using the values of the four metrics and their attachment type. The Tukey statistical test (confidence interval = 0.95; TukeyHSD from the stats package in R) was used on a simple linear model (separately between one of the four specified network metrics and the attachment type) to identify significant differences in mean values among the three categories of attachment type.

Using the constructed network (Figure 3), we further investigated edge‐level patterns. We tested for the presence and absence of associations being different from random chance (based on the number of host fish in a particular network and a particular trait‐based category) to test for host preference and niche partitioning among different classes of parasite attachment by taking the traits of host fish into account. To simulate random chance, we build 1000 null networks with a reshuffling of edges between the two classes of nodes while keeping the degree constrained (to account for specialisation of parasites and susceptibility of hosts) using the package *bipartite* (Dormann et al., 2009). To quantify how different the empirical frequency of a type of interaction (such as testing whether branchial parasites prefer schooling fish or non‐schooling fish in a marine environment), we performed the following series of computations: (1) we took the total frequency of interactions in each category (e.g., branchial parasites interacting with schooling fish) across all the null networks, (2) then we compared where the empirical values fall on the respective distribution (e.g., difference between schooling and non‐schooling fish impacted by buccal parasites) from all the null model values using a Z‐test (after testing for normality using a Shapiro–Wilk test) (see Swain et al., 2022), (3) then we combined the associated p‐values using Stouffer's method where necessary (i.e., multiple tests were being combined to make an inference), (4) we further correct for multiple correlations (tests for multiple categories) across all the categories using Benjamini‐Hochberg method.

## 3 RESULTS

### 3.1 Sample completeness

Using the abundance of individual parasite species, we found the parasite species have a species coverage of 0.9959 with an estimated richness (Hill number *q* = 0) of 21.996 (observed species richness = 21). The sampling coverage of interactions (where we used the occurrences of a parasite on a given host as the ‘abundance’ value in the iNEXT package) was 0.9751 (for more information, refer to Figure S1).

### 3.2 Skin‐attaching parasites are generalists

**FIGURE 4:**
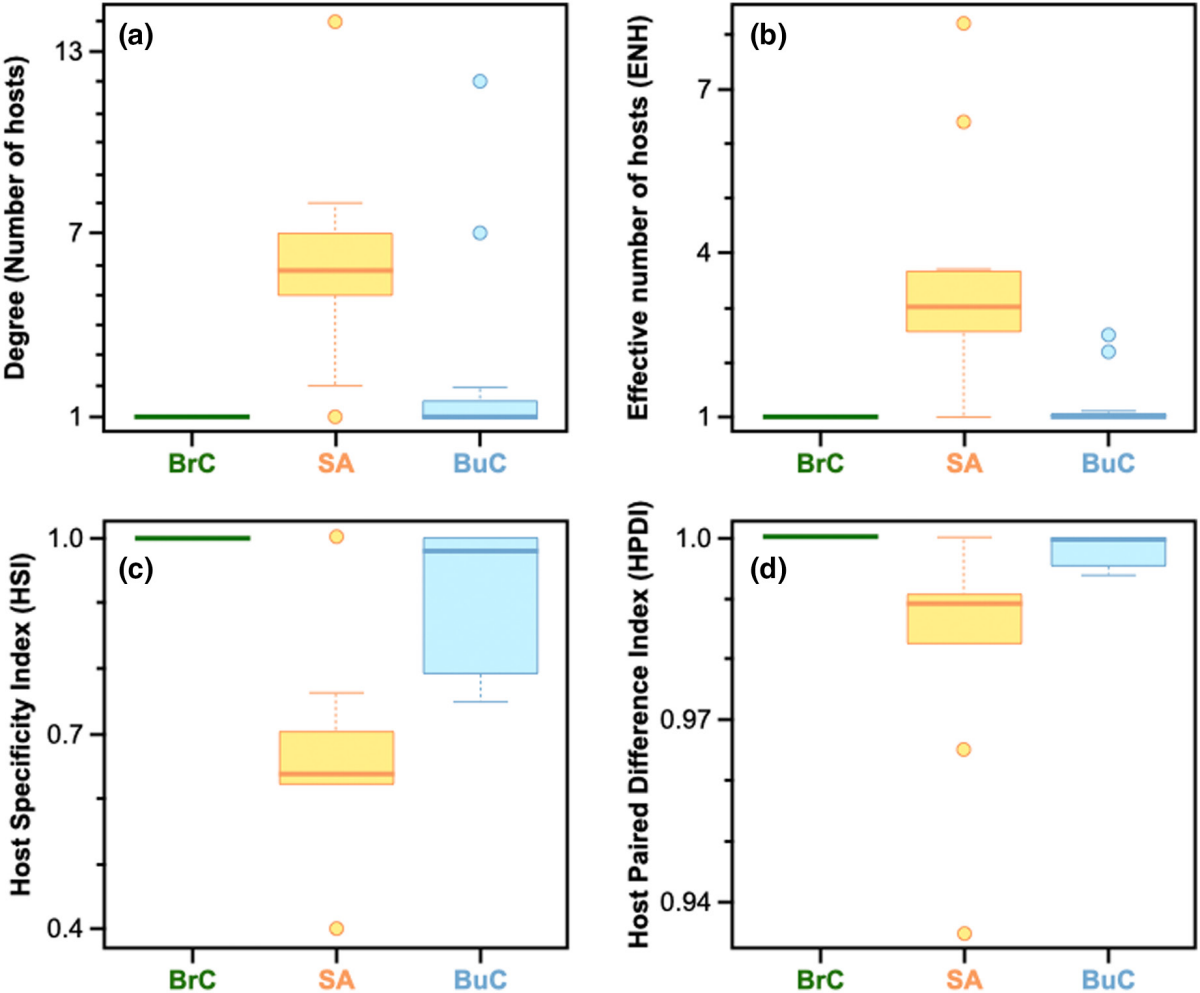
Comparisons of network metrics: Boxplot representations of (a) degree, (b) Effective number of hosts (ENH), (c) host specificity index (HSI), and (d) host paired difference index (HPDI) among isopod parasites (attachment site: BrC: branchial cavity, BuC: Buccal Cavity, SA: Skin Attaching). Branchial (attaching) parasites were found to be highly specialised when compared w skin‐attaching ones parasites in all the four metrics (degree: *p*‐adj < .05; ENH: *p*‐adj < .01; HSI: *p*‐adj < .01; HDPI: *p*‐adj < .05). Between buccal parasites and skin‐attaching ones, degree was not significantly different, difference in HSI was significant (*p*‐adj < .05) and that of ENH and HPDI were marginally significant (*p*‐adj < .1, but >.05). There were no significant differences between the buccal and branchial cavity parasites for any metric.

Based on our Tukey HSD results corrected for multiple correlations, skin (attaching) parasites were found to be more generalised than the other two classes of attachment types (Buccal and Branchial) due to higher values of degree, and ENH, and lower values of HSI and HPDI (see Figure 4 and Tables S1 and S2). Branchial (attaching) parasites were found to be highly specialised when compared with skin‐attaching parasites in all four metrics (degree: *p*‐adj < .05; ENH: *p*‐adj < .01; HSI: *p*‐adj < .01; HDPI: *p*‐adj < .05). However, this was not the case when the buccal parasites were compared with the skin‐attaching ones. The degree was not found to be significantly different between the two groups, but the difference in HSI was significant (*p* < .05) and that of ENH and HPDI were marginally significant (*p* < .1, but >.05). This signifies that even if the total number of hosts impacted by the buccal parasites is not significantly different from the skin‐attaching ones, the extent to which buccal ones impact each of the hosts is more skewed than skin‐attaching ones (i.e., buccal parasites impact similar number of hosts (degree) as the skin‐attaching ones, but primarily impact only a very small proportion of them when compared with skin‐attaching ones (evident in higher HSI for buccal ones)). There were no significant differences between the buccal and branchial cavity parasites (Figure 4). Detailed results from the models are in the Tables S1 and S2.

### 3.3 Niche partitioning of parasites by fish traits

Using the link‐level analysis across networks and the relative proportion of links between certain categories of fish hosts (as compared with null models) we found the following patterns (see Table S4 for details):

1. in marine environments, the branchial cavity‐attaching parasitic isopods were found to preferentially parasitise the pelagic, schooling fishes (deduced from the following results: pelagic fishes have disproportionately more branchial links in the marine environment, (*p*‐adj. < .05) and that the branchial parasites prefer schooling fish (*p*‐adj < .05));
2. the buccal cavity‐dwelling parasites affect both pelagic and demersal fishes (no difference from null; *p* > .05), but they preferentially parasitise non‐schooling fishes (adj. *p* < .01);
3. in a marine‐brackish water environment, the branchial cavity‐attaching parasites preferentially infect schooling fishes of the pelagic region (adj. *p* < .05) but have no preference among demersal schooling or non‐schooling fishes (adj. *p* > .05);
4. buccal cavity‐attaching parasites preferentially infect non‐schooling fishes of the demersal region in both marine and brackish‐marine environments (as compared with schooling fish, adj. *p* < .05) and in pelagic region in the brackish‐marine realm (as compared with schooling fish, adj. *p* < .05).
5. The patterns in skin‐attaching parasites were indistinguishable from null models.

## 4 DISCUSSION

Assessing whether traits of parasites (host attachment type) and host fish (habitat type, habitat salinity, and schooling) impact the presence or absence of interactions is important in predicting and understanding future interactions and the general structure of fish‐isopod parasite interactions in specific, and host–parasite systems in general. We found that the degree of specialisation (measured through network metrics) among Cymothoid parasites is associated with their attachment type: skin‐attaching parasites were found to be better generalists than others (Figure 4). Past literature suggests that they may have turned into generalists because of fewer environmental requirements and later diverged and started attaching to the skin in different regions of the body surface (Morton, 1974) due to a variety of available niches (please note that although skin‐attaching parasites are more generalised than the other two groups, it does not indicate they are indiscriminate in host choice).

There are two highly generalised buccal‐attaching parasites *Cymothoa indica* and *Catoessa boscii*. *Cymothoa indica* impacts fishes from different orders in both Odisha and West Bengal, but all of them are shallow, demersal feeders (Figure 3), and we hypothesise that the generalisation results from attachment to hosts during their feeding bouts. Similarly, even though *Catoessa boscii* affects 7 fish species (Figure 1), most of them have a very similar body structure (i.e. laterally compressed and deep‐bodied; Fischer & Bianchi, 1984) and 4 of them are in the same order (Carangiformes). In addition, even if these two buccal parasites impact a similar number of hosts (degree) as many skin‐attaching ones, they primarily impact only a small proportion of the hosts when compared with skin‐attaching ones (evident in higher HSI and lower ENH for these buccal parasites compared with skin‐attaching parasites; see Figure 4, Table S3).

The preference of branchial cavity‐dwelling parasites impacting schooling fish (in pelagic regions) might have to do with host density and ease of transfer (higher encounter rates) when the larvae are released from female parasites on an infected host. Past studies have shown that organisms that live in groups are more prone to parasite infection (Ezenwa, 2004) and that branchial attachment is the ancestral state among Cymothoid isopod parasites (Hata et al., 2017), therefore, a schooling‐based, pelagic fish might be the ancestral host in the system.

The preference of buccal cavity‐attaching parasite species to affect only non‐schooling fishes (seen strongly in demersal region and weakly in the pelagic realm), might be a result of restricting the competition with branchial cavity‐attaching species. The branchial parasites and larval stages need the gill/branchial region for residence (Williams & Bunkley‐Williams, 2019). There might have been a general change in the strategy of parasite dispersal from density dependence in schools (in branchial parasites) to density dependence during foraging or shared habitat (in buccal parasites) (e.g., ticks attack the hosts which use grassy regions as foraging areas (Richardson et al., 2022)), especially in the demersal region and shallow waters. This may lead to parasite transfer due to shared food habits or habitat with other hosts and possibly, new host invasions. For example, *Cymothoa indica*, after diverging from its sister groups, might have started residing in the benthic region and impacted the phylogenetically unrelated demersal fishes, most of which do not possess any other branchial parasites–leading to its intense host generalisation. However, future research is warranted to explore these hypotheses using larger datasets from multiple ecosystems.

**FIGURE 5:**
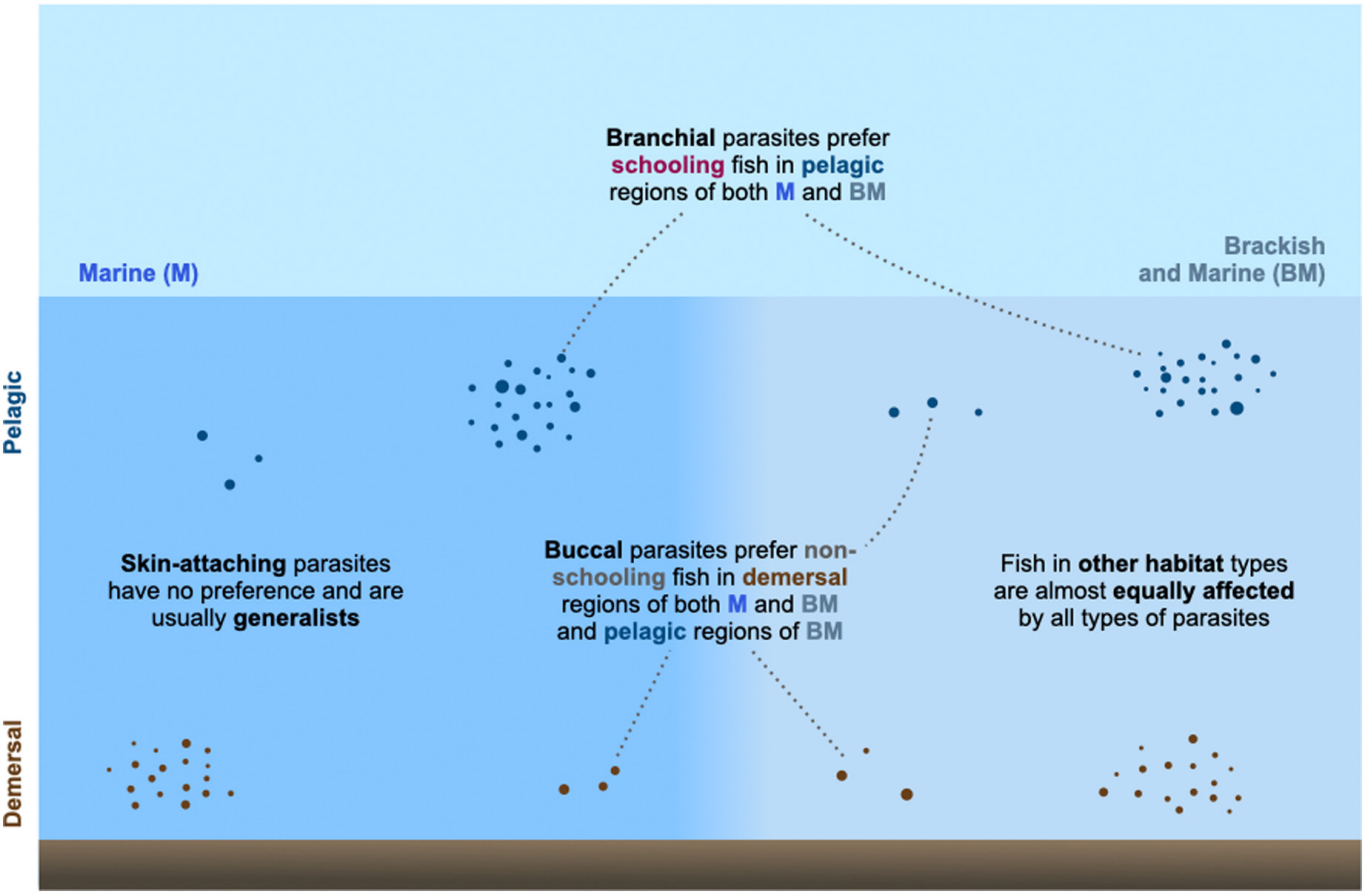
Niche partitioning and preferences: Conceptual figure of preferences of isopod parasites with different attachment types towards fish with different ecological traits. Other habitat types include the ones not mentioned in the figure, such as marine, pelagic, and non‐schooling; marine, demersal, and schooling; and brackish‐marine, demersal, and schooling. Please note that the larger number of fish in a group denotes schooling fish and the smaller number denotes non‐schooling fish; the sizes of the dots do not denote anything.

Overall, our work serves as a first step towards understanding what traits, of the parasite and their host fish, impact the preference and degree of parasitic association (Figure 5). Through our trait‐based and system‐level approaches, we hope that our study will aid in generating general trends and hypotheses in the host fish‐parasitic isopod system. Cymothoid isopods are generally less studied despite their global distribution and importance to fisheries (Smit et al., 2014); detailed phylogenetic and ecological studies across more ecosystems are necessary to validate our findings and further investigate patterns in these important fish–parasite associations.

## AUTHOR CONTRIBUTIONS

**Sandeep Kumar Mohapatra:** Conceptualization (equal); data curation (equal); formal analysis (equal); resources (equal); visualization (equal); writing – original draft (equal). **Anshuman Swain:** Conceptualization (equal); formal analysis (equal); methodology (equal); software (equal); visualization (equal); writing – original draft (equal); writing – review and editing (equal). **Dipanjan Ray:** Data curation (equal); supervision (equal). **Rajesh Kumar Behera:** Conceptualization (equal); writing – original draft (equal). **Basudev Tripathy:** Supervision (equal); writing – review and editing (equal). **Jaya Kishor Seth:** Conceptualization (equal); supervision (equal); writing – review and editing (equal). **Anil Mohapatra:** Formal analysis (equal); supervision (equal); writing – review and editing (equal).

## CONFLICT OF INTEREST STATEMENT

The authors declare no competing interests.

## Supporting information


Data S1.

## Data Availability

All data needed to repeat the results in this manuscript are available on https://github.com/anshuman21111/fish‐isopod‐parasite
