# Supplementary material for: Niche partitioning and host specialisation in fish‐parasitising isopods: Trait‐dependent patterns from three ecosystems on the east coast of India
Source: Ecol Evol. 2024 Sep 11;14(9):e70298. doi: 10.1002/ece3.70298 (PMC11390490; doi:10.1002/ece3.70298)
Supplement: Supplementary file 1 — Data S1. [file ECE3-14-e70298-s001.docx]

**Supplemental Figures
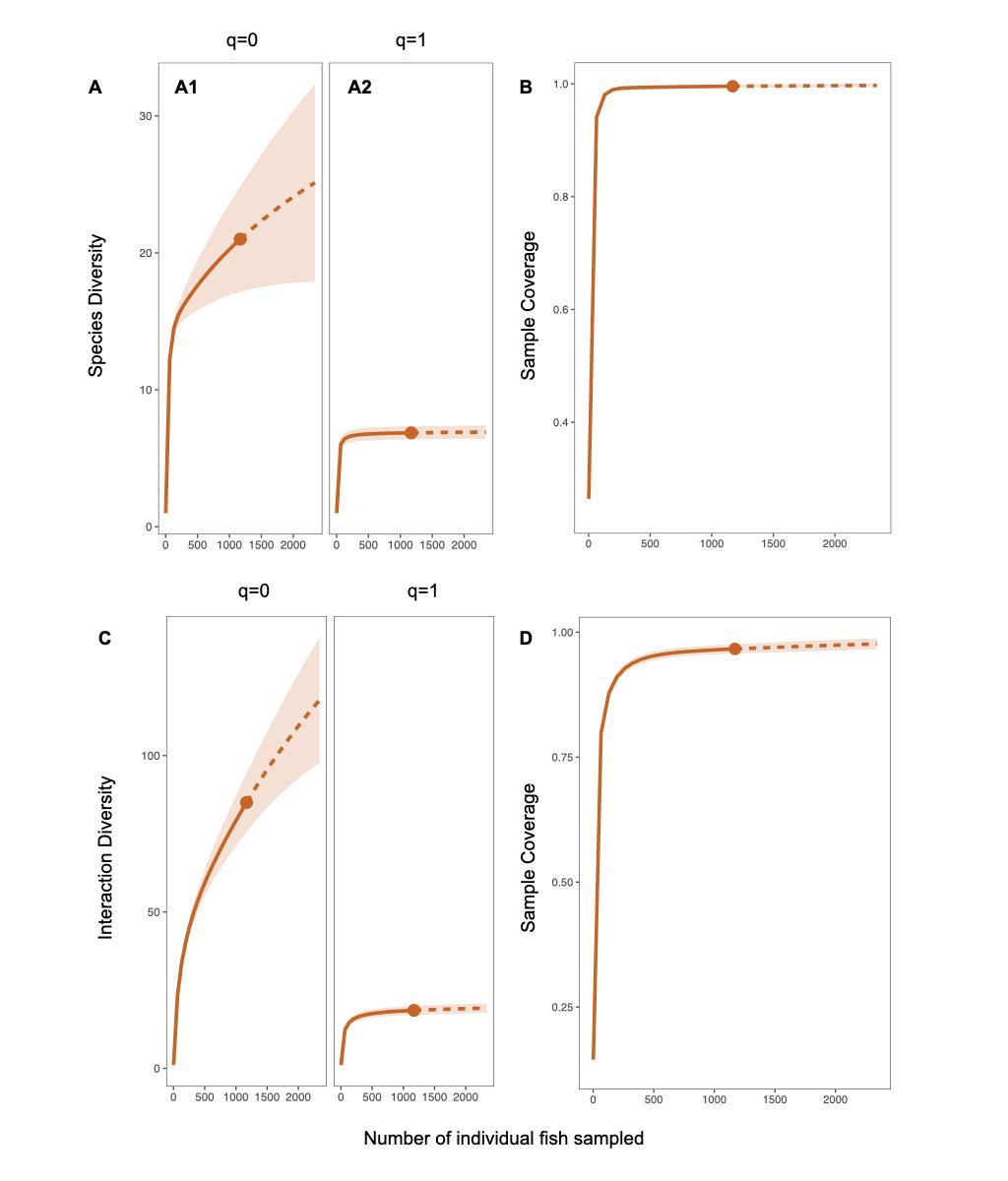
**

**Supplementary Figure 1. Sample completeness:** Rarefaction (solid lines) and extrapolated (dashed lines) plots for diversity and sample completeness for species (A and B) and interactions (C and D). A1 and C1 depict Hill number q=0 (richness) while A2 and C2 depict q=1 (scaled Shannon diversity). The results from q=1 and sample coverage indicate that the dominant/important species and interactions in the assembalge are already sampled. The solid dot represents the total sample size (of the number of individual fish sampled). All plots were produced using the iNEXT package in R.**Supplementary Table** **1**: Results from the analysis of variance for each of the four linear models (Network metrics regressed against the mode of attachment).

| **AOV model of Mode of Attachment with:** | **Df** | **Sum. Sq.** | **Mean Sq.** | **F Value** | **Pr(>F)** |
| --- | --- | --- | --- | --- | --- |
| Degree | 2 | 104.9 | 52.43 | 4.579 | 0.0247 |
| ENH (Eff. No. of Hosts) | 2 | 31.96 | 15.981 | 6.777 | 0.0064 |
| HSI (Host Specificity Index) | 2 | 0.5315 | 0.26573 | 14.57 | 0.0002 |
| HPDI (Host Paired Diff. Index) | 2 | 0.001696 | 0.0008478 | 4.787 | 0.0215 |

**Supplementary Table** **2**: Pairwise comparisons of four network metrics for each pair of attachment types using a Tukey HSD test (on a linear model between the network metric and the attachment site; see methods for details) showing the 95% confidence intervales (difference mean, the lower and upper bounds) and associated p-values.

1. **For Degree:**

| **Comparison** | **Difference** | **Lower** | **Upper** | **p-adj** |
| --- | --- | --- | --- | --- |
| SA-Br | 5.111111 | 0.7591081 | 9.463114 | 0.0201113 |
| BuC-Br | 3.600000 | 1.4565705 | 8.656571 | 0.1923670 |
| BuC-SA | -1.511111 | -6.3278958 | 3.305674 | 0.7073845 |

1. **For ENH** (Effective No. of Hosts)**:**

| **Comparison** | **Difference** | **Lower** | **Upper** | **p-adj** |
| --- | --- | --- | --- | --- |
| SA-Br | 2.6937480 | 0.7186063 | 4.66888982 | 0.0071562 |
| BuC-Br | 0.5717465 | -1.7231609 | 2.86665387 | 0.8025338 |
| BuC-SA | -2.1220015 | -4.3080829 | 0.06407982 | 0.0579428 |

1. **For HSI** (Host Specificity Index)**:**

| **Comparison** | **Difference** | **Lower** | **Upper** | **p-adj** |
| --- | --- | --- | --- | --- |
| SA-Br | -0.35329900 | -0.52696127 | -0.1796367 | 0.0001732 |
| BuC-Br | -0.09629546 | -0.29807279 | 0.1054819 | 0.4581820 |
| BuC-SA | 0.25700354 | 0.06479462 | 0.4492125 | 0.0082944 |

1. **For HPDI** (Host Paired Diff. Index)**:**

| **Comparison** | **Difference** | **Lower** | **Upper** | **p-adj** |
| --- | --- | --- | --- | --- |
| SA-Br | -0.019014502 | -0.036130356 | -0.001898648 | 0.0281809 |
| BuC-Br | -0.002245327 | -0.022132153 | 0.017641498 | 0.9553734 |
| BuC-SA | 0.016769175 | -0.002174605 | 0.035712954 | 0.0880317 |

**Supplementary Table** **3**: Summary values for each parasite species.

| **Parasite** | **Region of Attachment** | **degree**  **(No. of hosts)** | **HSI** | **HPDI** | **ENH** | **No. of host fish families** |
| --- | --- | --- | --- | --- | --- | --- |
| *Agarna malayi* | Br | 1 | 1 | 1 | 1 | 1 |
| *Joryma hilsae* | Br | 1 | 1 | 1 | 1 | 1 |
| *Joryma malabaricus* | Br | 1 | 1 | 1 | 1 | 1 |
| *Joryma sawayah* | Br | 1 | 1 | 1 | 1 | 1 |
| *Mothocya colletei* | Br | 1 | 1 | 1 | 1 | 1 |
| *Mothocya renardi* | Br | 1 | 1 | 1 | 1 | 1 |
| *Norileca indica* | Br | 1 | 1 | 1 | 1 | 1 |
| *Catoessa boscii* | BuC | 7 | 0.793 | 0.995 | 2.213 | 3 |
| *Cymothoa eremita* | BuC | 1 | 1 | 1 | 1 | 1 |
| *Cymothoa frontalis* | BuC | 2 | 0.980 | 0.999 | 1.103 | 1 |
| *Cymothoa indica* | BuC | 12 | 0.746 | 0.994 | 2.542 | 10 |
| *Lobothorax typus* | BuC | 1 | 1 | 1 | 1 | 1 |
| *Anilocra dimidiata* | SA | 7 | 0.636 | 0.989 | 3.668 | 5 |
| *Nerocila depressa* | SA | 5 | 0.676 | 0.990 | 2.796 | 3 |
| *Nerocila loveni* | SA | 5 | 0.621 | 0.983 | 3.039 | 1 |
| *Nerocila orbignyi* | SA | 2 | 0.701 | 0.983 | 2 | 2 |
| *Nerocila phaiopleura* | SA | 14 | 0.399 | 0.965 | 8.200 | 14 |
| *Nerocila poruvae* | SA | 7 | 0.761 | 0.994 | 2.581 | 6 |
| *Nerocila serra* | SA | 8 | 0.396 | 0.935 | 6.395 | 5 |
| *Nerocila sigani* | SA | 6 | 0.630 | 0.989 | 3.564 | 4 |
| *Nerocila sundaica* | SA | 1 | 1 | 1 | 1 | 1 |

**Supplementary Table** **4**: Statistical metrics for each hypothesis explicitly tested for trait-dependence of parasite attachment and noted in the manuscript. Please note that X vs. Y generally translates to their difference in the test.

| **Hypothesis and Test** | **Z-score** of the empirical value w.r.t null dist. | **p-value** associated with Z-test | **W-statistic** of the null distribution (Shapiro-Wilk test) | **p-value** associated with the Shapiro-Wilk test |
| --- | --- | --- | --- | --- |
| Pelagic fishes have more branchial links in marine environment  (Test: number of fish species that have branchial links vs. others in marine environment) | 1.992 | 0.0463 | 0.928 | 0.4351 |
| Branchial parasites prefer schooling fish  (Test: number of schooling vs. non-schooling fish species impacted by branchial species) | 1.966 | 0.0493 | 0.942 | 0.6342 |
| Buccal cavity-dwelling parasites affect demersal fishes more  (Test: number of demersal vs. pelagic fish species impacted by buccal species) | 0.912 | 0.3620 | 0.977 | 0.5317 |
| Buccal cavity-dwelling parasites preferentially impact non-schooling fishes  (Test: number of schooling vs. non-schooling fish species impacted by buccal species) | 2.731 | 0.0063 | 0.876 | 0.4521 |
| Pelagic fishes have more branchial links in marine-brackish environment  (Test: number of fish species that have branchial links vs. others in marine-brackish environment) | 2.012 | 0.0442 | 0.968 | 0.8250 |
| Schooling fishes have more branchial links in marine-brackish environment  (Test: number of schooling vs. non-schooling fish species impacted by branchial species in marine-brackish env.) | 1.639 | 0.1013 | 0.841 | 0.4019 |
| Non-schooling fishes have more buccal links in demersal marine-brackish environment  (Test: number of schooling vs. non-schooling fish species impacted by branchial species in demersal marine-brackish env.) | 2.056 | 0.0398 | 0.883 | 0.2026 |
| Non-schooling fishes have more buccal links in demersal marine environment  (Test: number of schooling vs. non-schooling fish species impacted by branchial species in demersal marine env.) | 2.237 | 0.0253 | 0.887 | 0.1289 |
| Non-schooling fishes have more buccal links in pelagic marine-brackish environment  (Test: number of schooling vs. non-schooling fish species impacted by branchial species in pelagic marine-brackish env.) | 2.043 | 0.0411 | 0.916 | 0.2527 |

**Supplementary Table** 5: List of Fishes examined for the presence of parasitic Isopods.

| **Sl. No.** | **Fish name** | **Parasite found/ not found** | **No. of fish checked** | **No. of fish individuals found affected by Parasite** |
| --- | --- | --- | --- | --- |
| 1 | *Thryssa dussumieri* (Valenciennes, 1848) | Found | 460 | 23 |
| 2 | *Terapon puta* Cuvier, 1829 | Found | 540 | 1 |
| 3 | *Terapon jarbua* (Forsskål, 1775) | Found | 102 | 8 |
| 4 | *Strongylura strongylura* (Hasselt 1823) | Found | 216 | 48 |
| 5 | *Strongylura leiura* (Bleeker, 1850) | Found | 126 | 26 |
| 6 | *Strophidon sathete* (Hamilton, 1822) | Found | 32 | 1 |
| 7 | *Siganus javus* (Linnaeus, 1766) | Found | 172 | 27 |
| 8 | *Siganus canaliculatus* (Park, 1797) | Found | 436 | 29 |
| 9 | *Setipinna taty* (Valenciennes, 1848) | Found | 92 | 15 |
| 10 | *Secutor insidiator* (Bloch, 1787) | Found | 124 | 12 |
| 11 | *Selaroides leptolepis* (Cuvier, 1833) | Found | 164 | 6 |
| 12 | *Sardinella longiceps* Valenciennes, 1847 | Found | 653 | 35 |
| 13 | *Sardinella gibbosa* (Bleeker, 1849) | Found | 1065 | 28 |
| 14 | *Rhynchorhamphus georgii* (Valenciennes, 1847) | Found | 17 | 1 |
| 15 | *Rastrelliger kanagurta* (Cuvier, 1816) | Found | 648 | 19 |
| 16 | *Priacanthus tayenus* Richardson 1846 | Found | 91 | 16 |
| 17 | *Plotosus lineatus* (Thunberg, 1787) | Found | 62 | 12 |
| 18 | *Plotosus canius* Hamilton, 1822 | Found | 47 | 2 |
| 19 | *Parastromateus niger* (Bloch, 1795) | Found | 128 | 6 |
| 20 | *Oreochromis mossambicus* (Peters, 1852) | Found | 562 | 23 |
| 21 | *Opisthopterus tardoore* (Cuvier, 1829) | Found | 74 | 8 |
| 22 | *Nibea maculata* (Bloch & Schneider, 1801) | Found | 89 | 6 |
| 23 | *Nemipterus japonicus* (Bloch, 1791) | Found | 105 | 11 |
| 24 | *Nematalosa nasus* (Bloch, 1795) | Found | 543 | 19 |
| 25 | *Mugil cephalus* Linnaeus, 1758 | Found | 287 | 2 |
| 26 | *Lutjanus lutjanus* Bloch, 1790 | Found | 51 | 1 |
| 27 | *Lutjanus johnii* (Bloch, 1792) | Found | 77 | 1 |
| 28 | *Lobotes surinamensis* (Bloch, 1790) | Found | 76 | 3 |
| 29 | *Lepturacanthus savala* (Cuvier, 1829) | Found | 265 | 25 |
| 30 | *Leiognathus splendens* (Cuvier, 1829) | Found | 61 | 7 |
| 31 | *Leiognathus longispinis* (Valenciennes, 1835) | Found | 476 | 6 |
| 32 | *Leiognathus blochi* (Valenciennes, 1835) | Found | 97 | 12 |
| 33 | *Lates calcarifer* (Bloch, 1790) | Found | 163 | 25 |
| 34 | *Lactarius lactarius* (Bloch & Schneider 1801) | Found | 25 | 1 |
| 35 | *Karalla daura* (Cuvier, 1829) | Found | 265 | 7 |
| 36 | *Johnius dussumieri* (Cuvier, 1830) | Found | 132 | 8 |
| 37 | *Johnius carutta* Bloch, 1793 | Found | 152 | 6 |
| 38 | *Ilisha melastoma* (Bloch & Schneider, 1801) | Found | 124 | 2 |
| 39 | *Glossogobius giuris* (Hamilton, 1822) | Found | 652 | 98 |
| 40 | *Gerres oyena* (Forsskål, 1775) | Found | 312 | 4 |
| 41 | *Eubleekeria splendens* (Cuvier, 1829) | Found | 476 | 21 |
| 42 | *Etroplus suratensis* (Bloch, 1790) | Found | 243 | 56 |
| 43 | *Escualosa thoracata* (Valenciennes, 1847) | Found | 453 | 9 |
| 44 | *Epinephelus coioide*s (Hamilton, 1822) | Found | 49 | 2 |
| 45 | *Dussumieria acuta* (Valenciennes, 1847) | Found | 176 | 17 |
| 46 | *Deveximentum insidiato*r (Bloch 1787) | Found | 472 | 12 |
| 47 | *Datnioides polota* (Hamilton, 1822) | Found | 367 | 67 |
| 48 | *Chirocentrus nudus* Swainson, 1839 | Found | 143 | 1 |
| 49 | *Carangoides malabaricus* (Bloch & Schneider, 1801) | Found | 104 | 20 |
| 50 | *Carangoides ferdau* (Forsskål, 1775) | Found | 125 | 3 |
| 51 | *Arius maculatus* (Thunberg, 1792) | Found | 32 | 1 |
| 52 | *Arius arius* (Hamilton, 1822) | Found | 97 | 1 |
| 53 | *Ambassis ambassis* (Lacepède, 1802) | Found | 457 | 28 |
| 54 | *Alepes kleinii* (Bloch, 1793) | Found | 143 | 16 |
| 55 | *Alepes djedaba* (Forsskål, 1775) | Found | 148 | 51 |
| 56 | *Acanthopagrus berda* (Forsskål, 1775) | Found | 154 | 19 |
| 57 | *Ablennes hians* (Valenciennes, 1846) | Found | 9 | 1 |
| 58 | *Dussumieria acuta* (Valenciennes, 1847) | Not found | 146 | 0 |
| 59 | *Anodontostoma chacunda* (Hamilton, 1822) | Not found | 54 | 0 |
| 60 | *Hilsa kelee* (Cuvier, 1829) | Not found | 121 | 0 |
| 61 | *Sardinella fimbriata* (Valenciennes, 1847) | Not found | 97 | 0 |
| 62 | *Tenualosa toli* (Valenciennes, 1847) | Not found | 75 | 0 |
| 63 | *Stolephorus commersonnii* Lacepède, 1803 | Not found | 46 | 0 |
| 64 | *Thryssa hamiltonii* Gray, 1835 | Not found | 134 | 0 |
| 65 | *Thryssa mystax* (Bloch & Schneider, 1801) | Not found | 165 | 0 |
| 66 | *Chirocentrus dorab* (Forsskål, 1775) | Not found | 76 | 0 |
| 67 | *Thryssa purava* (Hamilton, 1822) | Not found | 64 | 0 |
| 68 | *Thryssa kammalensis* (Bleeker, 1849) | Not found | 162 | 0 |
| 69 | *Arius gagora* (Hamilton, 1822) | Not found | 45 | 0 |
| 70 | *Netuma thalassina* (Rüppell, 1837) | Not found | 167 | 0 |
| 71 | *Plicofollis dussumieri* (Valenciennes, 1840) | Not found | 62 | 0 |
| 72 | *Harpadon nehereus* (Hamilton, 1822) | Not found | 79 | 0 |
| 73 | *Saurida micropectoralis* Shindo & Yamada, 1972 | Not found | 84 | 0 |
| 74 | *Saurida tumbi*l (Bloch, 1795) | Not found | 68 | 0 |
| 75 | *Saurida undosquamis* (Richardson, 1848) | Not found | 93 | 0 |
| 76 | *Trachinocephalus trachinus* (Temminck & Schlegel, 1846) | Not found | 67 | 0 |
| 77 | *Myripristis murdjan* (Forsskål, 1775) | Not found | 54 | 0 |
| 78 | *Sargocentron rubrum* (Forsskål, 1775) | Not found | 74 | 0 |
| 79 | *Scomberomorus guttatus* (Bloch & Schneider, 1801) | Not found | 38 | 0 |
| 80 | *Pampus chinensis* (Euphrasen, 1788) | Not found | 76 | 0 |
| 81 | *Fistularia petimba* Lacepède, 1803 | Not found | 83 | 0 |
| 82 | *Upeneus sulphureus* Cuvier, 1829 | Not found | 137 | 0 |
| 83 | *Parupeneus indicus* (Shaw, 1803) \| Species | Not found | 75 | 0 |
| 84 | *Upeneus vittatus* (Forsskål, 1775) | Not found | 92 | 0 |
| 85 | *Ostorhinchus fasciatus* (White, 1790) | Not found | 58 | 0 |
| 86 | *Jaydia poeciloptera* (Cuvier, 1828) | Not found | 83 | 0 |
| 87 | *Ostorhinchus fleurieu* Lacepède, 1802 | Not found | 69 | 0 |
| 88 | *Kurtus indicus* Bloch, 1786 | Not found | 54 | 0 |
| 89 | *Butis butis* (Hamilton, 1822) | Not found | 82 | 0 |
| 90 | *Butis koilomatodon* (Bleeker, 1849) | Not found | 49 | 0 |
| 91 | *Eleotris melanosoma* Bleeker, 1853 | Not found | 126 | 0 |
| 92 | *Boleophthalmus boddarti* (Pallas, 1770) | Not found | 36 | 0 |
| 93 | *Oligolepis acutipennis* (Valenciennes, 1837) | Not found | 183 | 0 |
| 94 | *Parachaeturichthys polynema* (Bleeker, 1853) | Not found | 143 | 0 |
| 95 | *Trypauchen vagina* (Bloch & Schneider, 1801) | Not found | 192 | 0 |
| 96 | *Alectis indica* (Rüppell, 1830) | Not found | 64 | 0 |
| 97 | *Atropus atropos* (Bloch & Schneider, 1801) | Not found | 153 | 0 |
| 98 | *Caranx sexfasciatus* Quoy & Gaimard, 1825 | Not found | 184 | 0 |
| 99 | *Decapterus russelli* (Rüppell, 1830) | Not found | 153 | 0 |
| 100 | *Scomberoides lysan* (Forsskål 1775) | Not found | 253 | 0 |
| 101 | *Seriolina nigrofasciata* (Rüppell, 1829) | Not found | 93 | 0 |
| 102 | *Lactarius lactarius* (Bloch & Schneider, 1801) | Not found | 95 | 0 |
| 103 | *Coryphaena hippurus* Linnaeus, 1758 | Not found | 84 | 0 |
| 104 | *Rachycentron canadum* (Linnaeus, 1766) | Not found | 54 | 0 |
| 105 | *Mene maculata* (Bloch & Schneider, 1801) | Not found | 93 | 0 |
| 106 | *Eleutheronema tetradactylum* (Shaw, 1804) | Not found | 74 | 0 |
| 107 | *Filimanus xanthonema* (Valenciennes, 1831) | Not found | 52 | 0 |
| 108 | *Polydactylus plebeius* (Broussonet, 1782) | Not found | 34 | 0 |
| 109 | *Polydactylus sextarius* (Bloch & Schneider, 1801) | Not found | 97 | 0 |
| 110 | *Sphyraena putnamae* Jordan & Seale, 1905 | Not found | 36 | 0 |
| 111 | *Hyporhamphus limbatus* (Valenciennes, 1847) | Not found | 136 | 0 |
| 112 | *Rhynchorhamphus georgii* (Valenciennes, 1847) | Not found | 172 | 0 |
| 113 | *Tylosurus crocodilus* (Péron & Lesueur, 1821) | Not found | 137 | 0 |
| 114 | *Osteomugil speigleri* (Bleeker, 1858) | Not found | 213 | 0 |
| 115 | *Planiliza macrolepis* (Smith, 1846) | Not found | 314 | 0 |
| 116 | *Drepane longimana* (Bloch & Schneider, 1801) | Not found | 154 | 0 |
| 117 | *Ephippus orbis* (Bloch, 1787) | Not found | 94 | 0 |
| 118 | *Platax teira* Forsskål, 1775 | Not found | 145 | 0 |
| 119 | *Ephippus orbis* (Bloch, 1787) | Not found | 172 | 0 |
| 120 | Equulites leuciscus (Günther, 1860) | Not found | 231 | 0 |
| 121 | *Gazza minuta* (Bloch, 1795) | Not found | 153 | 0 |
| 122 | *Gazza dentex* (Valenciennes, 1835) | Not found | 176 | 0 |
| 123 | *Acanthurus mata* (Cuvier, 1829) | Not found | 132 | 0 |
| 124 | *Lutjanus fulviflamma* (Forsskål, 1775) | Not found | 91 | 0 |
| 125 | *Lutjanus malabaricus* (Bloch & Schneider, 1801) | Not found | 89 | 0 |
| 126 | *Pinjalo pinjalo* (Bleeker, 1850) | Not found | 74 | 0 |
| 127 | *Gerres filamentosus* Cuvier, 1829 | Not found | 54 | 0 |
| 128 | *Diagramma pictum* (Thunberg, 1792) | Not found | 74 | 0 |
| 129 | *Pomadasys kaakan* (Cuvier, 1830) | Not found | 43 | 0 |
| 130 | *Pomadasys maculatus* (Bloch, 1793) | Not found | 76 | 0 |
| 131 | *Rhabdosargus sarba* (Forsskål, 1775) | Not found | 97 | 0 |
| 132 | *Scolopsis vosmer*i (Bloch, 1792) | Not found | 54 | 0 |
| 133 | *Sillago sihama* (Forsskål, 1775) | Not found | 65 | 0 |
| 134 | *Protonibea diacanthus* (Lacepède, 1802) | Not found | 47 | 0 |
| 135 | *Aluterus monoceros* (Linnaeus, 1758) | Not found | 184 | 0 |
| 136 | *Chelonodontops patoca* (Hamilton, 1822) | Not found | 253 | 0 |
| 137 | *Arothron stellatus* (Anonymous, 1798) | Not found | 132 | 0 |
| 138 | *Lagocephalus lunaris* (Bloch & Schneider, 1801) | Not found | 312 | 0 |
| 139 | *Takifugu oblongus* (Bloch, 1786) | Not found | 247 | 0 |
| 140 | *Minous monodactylu*s (Bloch & Schneider, 1801) | Not found | 263 | 0 |
| 141 | *Grammoplites scaber* (Linnaeus, 1758) | Not found | 197 | 0 |
| 142 | *Platycephalus indicus* (Linnaeus, 1758) | Not found | 261 | 0 |
| 143 | *Ichthyscopus lebeck* (Bloch & Schneider, 1801) | Not found | 97 | 0 |
| 144 | *Uranoscopus crassiceps* Alcock, 1890 | Not found | 145 | 0 |
| 145 | *Pterois russelii* Bennett, 1831 | Not found | 182 | 0 |
